# Biocompatibility and Corrosion Protection Behaviour of Hydroxyapatite Sol-Gel-Derived Coatings on Ti6Al4V Alloy

**DOI:** 10.3390/ma10020094

**Published:** 2017-01-24

**Authors:** Amir A. El Hadad, Eduardo Peón, Federico R. García-Galván, Violeta Barranco, Juan Parra, Antonia Jiménez-Morales, Juan Carlos Galván

**Affiliations:** 1 Centro Nacional de Investigaciones Metalúrgicas (CSIC), Madrid 28040, Spain; amirelhadad@yahoo.com (A.A.E.H.); f.garciag@cenim.csic.es (F.R.G.-G.); 2 Biophysics Branch, Physics Department, Al-Azhar University, Nasr City, Cairo 11884, Egypt; 3 Centro de Biomateriales, Universidad de La Habana, Havana 10600, Cuba; epeon@biomat.uh.cu; 4 Instituto de Ciencia de Materiales de Madrid (CSIC), Madrid 28049, Spain; violeta.barranco@gmail.com; 5 Unidad de Investigación Clínica y Biopatología Experimental, Hospital Provincial de Ávila, Unidad Asociada al CSIC, Ávila 05003, Spain; jparra@ictp.csic.es; 6 Centro de Investigación Biomédica en Red en Bioingeniería, Biomateriales y Nanomedicina (CIBER-BBN), Madrid 28029, Spain; 7 Departamento de Ciencia e Ingeniería de Materiales e Ingeniería Química, Universidad Carlos III de Madrid, Leganés (Madrid) 28911, Spain; toni@ing.uc3m.es

**Keywords:** biomaterial, hydroxyapatite, calcium phosphate, sol-gel, Ti6Al4V alloy, corrosion protection, bioactivity, biocompatibility

## Abstract

The aim of this work was to prepare hydroxyapatite coatings (HAp) by a sol-gel method on Ti6Al4V alloy and to study the bioactivity, biocompatibility and corrosion protection behaviour of these coatings in presence of simulated body fluids (SBFs). Thermogravimetric/Differential Thermal Analyses (TG/DTA) and X-ray Diffraction (XRD) have been applied to obtain information about the phase transformations, mass loss, identification of the phases developed, crystallite size and degree of crystallinity of the obtained HAp powders. Fourier Transformer Infrared Spectroscopy (FTIR) has been utilized for studying the functional groups of the prepared structures. The surface morphology of the resulting HAp coatings was studied by Scanning Electron Microscopy (SEM). The bioactivity was evaluated by soaking the HAp-coatings/Ti6Al4V system in Kokubo’s Simulated Body Fluid (SBF) applying Inductively Coupled Plasma (ICP) spectrometry. 3-[4,5-dimethylthiazol-2-yl]-2,5-diphenyl tetrazolium bromide (MTT) and Alamar blue cell viability assays were used to study the biocompatibility. Finally, the corrosion behaviour of HAp-coatings/Ti6Al4V system was researched by means of Electrochemical Impedance Spectroscopy (EIS). The obtained results showed that the prepared powders were nanocrystalline HAp with little deviations from that present in the human bone. All the prepared HAp coatings deposited on Ti6Al4V showed well-behaved biocompatibility, good bioactivity and corrosion protection properties.

## 1. Introduction

Bone tissue is a natural composite material that comprises of both inorganic-hydroxyapatite (HAp)-and organic-collagen-constituents. The HAp has the formula Ca_10_(PO4)_6_(OH)_2_, amounts to 65% of the total bone mass, with the remaining mass formed by “mostly collagen” organic matter and water [1,2]. The collagen molecules are bonded together developing fibrils, which bound together to form fibres. Between these fibres there are small interstitial empty spaces, where apatite nanocrystals are deposited. The HAp seems to be the most appropriate ceramic material for hard-tissue replacement. HAp ceramics do not exhibit any cytotoxic effects, show excellent biocompatibility with hard tissues, skin and muscles tissues. For this reason, the most used calcium phosphate in implant fabrication is HAp, a biological active material with different forms, particles, films, coatings, fibres, biocomposites which has extensive biomedical applications [3,4]. Nano-sized HAp powder has a high specific surface area and therefore exhibits enhanced activity toward chemical and biological interactions in the human body. HAp has been known to formin vitro and in vivo bioactive bone-like apatite layer spontaneously on its surfaces [5,6]. The formed apatite layer acts as interface between the implant and tissue facilitating the formation of chemical and biological bond with the osseous tissue. Therefore, the essential prerequisite for a biomaterial to bond directly to living bone is the formation of an apatite layer on its surface when implanted in the body. Then the protein absorption onto the surface of such layer will trigger the osteoblasts, “new bone forming cells”, to differentiate, reproduce form new bone tissue.

The HAp can be synthesized by many methods [7,8]. The sol-gel synthesis of HA ceramics has attracted much attention because it offers a molecular-level mixing of the calcium and phosphorus precursors, which is capable of improving chemical homogeneity of the resulting HAp to a significant extent, in comparison with conventional methods such as solid state reactions, wet precipitation and hydrothermal synthesis [9,10,11,12]. The HAp synthesized by sol-gel will form strong chemical bonds with bone in vivo. All of these mentioned properties place the HAp into a class of biomaterials known as bioactive materials. Unfortunately, brittleness limits its usage for load bearing applications. For that it can only be used as bone filler and bone graft substitute in orthopaedics [13]. The only exception where HAp is applied in dynamically loaded situations is when it is used as a coating material on metallic materials. When metallic materials are implanted in the human body, some of the consequences of in vivo degradation are the metal ion release from the surface of the implanted material which can cause a harmful effect when escaping to the surrounding tissue [14]. Thus, the best implant metallic material will be the one exhibiting minimum ion release of less toxic ions and maximum biocompatibility. For this reason, the present work is aimed to design a multifunctional sol-gel-derived hydroxyapatite coating on Ti6Al4V surface. This coating must enhance the bioactivity and biocompatibility of the implant surface, as well as, avoid the metal ion release.

Nowadays the electrochemical methods for evaluation of barrier properties of coatings against the corrosion process, which promote metal ion release, are well established [15,16]. High ionic resistance of coatings is correlated with good corrosion protection behaviour and low ionic resistance, which is developed during exposure to aggressive environments, is correlated with poor protection behaviour [15]. Nevertheless, it is necessary to note that an aqueous aggressive medium like SBF is needed for the formation of an apatite layer on its surface when implanted in the body, but is also responsible of the corrosion process. The necessity of porous materials in biomedical applications has become mandatory as it plays an important role on the performance of implants via increasing the implant-tissue contact interface, thereby improving the bone implant integration. Both, bone repair and regeneration occurs along implant surface, and a coating with porous structures would also permit the attachment of biologically active molecules on these surfaces to improve the tissue response [17,18,19]. In terms of corrosion protection in a protective coating, the barrier action afforded by the coating requires the reduction of porosity to the lowest possible level. Obviously, the requirements for both, the bioactive and the protective mechanisms oppose each other.

At present, strong requirements in orthopaedics are still to be met; different approaches are needed to fulfil the challenges faced by this medical field. Concerning HAp coatings applied onto a biometallic surface, it can be found in literature scientific articles dealing with the design and optimum conditions for obtaining hydroxyapatite coating together with the evaluation of one of the many requirements that the implant surface must have to be able to be used in practice. That is, in most of the articles it is obtained a new coating and studied either their bioactivity, or their biocompatibility, or the release of toxic ions, or even less often their barrier properties and corrosion protection. However, nevertheless, in all of them, only one or two of these requirements are studied in depth, and at best, the coating design is combined with the study of bioactivity and biocompatibility, that is, three of them.

However, the present study is carried out by a multidisciplinary consortium of specialists that not only design, obtain, characterise and optimize the HAp coating to be applied onto Ti6Al4V, but also the evaluation of the bioactivity in SBF, biocompatibility in cell line of human fetal osteoblast, cytotoxicity, cell adhesion and proliferation, barrier properties and corrosion protection are studied. That is, all the requirements that the coating must fulfil, as a whole, and at the same time, to be able to be used in practical applications.

However, it is a great challenge to achieve a coating having good bioactivity and biocompatibility that, at the same time, provides good corrosion resistance and thus reduces the release of toxic ions. This is due to the fact that, in order to obtain good bioactivity and biocompatibility it is necessary to obtain a coating with an optimal porosity, which allows the entry of SBF for the generation of new bone and to serve as a scaffold providing a three-dimensional structure in which bone/implant adherence is optimal. However, porosity in a coating is a design variable that should be avoided when good barrier effect is required to prevent the release of toxic ions into body fluids.

To overcome the great challenge above exposed, an original methodology to obtain a multifunctional coating, which provides good bioactivity, biocompatibility and which reduce the release of toxic ions, is proposed. For this, a tailored designed coating that combines different degrees of porosity has been obtained. The outer layer has higher degree of porosity, which enhances the bioactivity and biocompatibility of the coating and the innermost layer, in contact with the metal surface, has higher degree of crosslinking and lower porosity, acting as a barrier and thus reducing the release of toxic ions. This is achieved by the iterative methodology proposed, in which the application of three layers with these special chemistry, dipping and withdraw speed, temperature and time provides the tailored coating. The intrinsic porosity of the inner layer is filled by the application of the second layer under these conditions. In addition, the second layer has a thermal stress absorbing role, reducing the mismatch due to the difference of the thermal expansion coefficients between metal and coating. It is also a stabiliser of the coating bulk integrity and structure, making possible to obtain a thicker coating without the internal stresses of the thick coatings obtained by other methodologies.

## 2. Experimental Section

### 2.1. Preparation of Hydroxyapatite Sol

Hydroxyapatite sols were obtained by hydrolysis and condensation of triethyl phosphite, (TEP) (C_6_H_5_O)_3_P (Sigma-Aldrich, St. Louis, MO, USA) and calcium nitrate tetrahydrate, Ca(NO_3_)_2_·4H_2_O (Panreac, Barcelona, Spain), following a variant of the sol-gel process described by Liu D.M. [9]. The hydrolysis of the phosphorus precursor (triethyl phosphite) was made by using anhydrous ethanol as solvent and adding a small amount of distilled water. The molar ratio of water to phosphorus precursor was fixed at 3. The mixture was introduced in a parafilm-sealed glass container and vigorously stirred for 30 min and then kept static at ambient temperature for 24 h. After that a stoichiometric amount (i.e., to maintain a Ca/P molar ratio 1.67) of 3 mol of Ca(NO_3_)_2_·4H_2_O was dissolved first in anhydrous ethanol and then added dropwise into the hydrolysed phosphite sol. The mixed solution was then continuously agitated for additional 30 min. The pH value was adjusted to be 7.5 and the sol was aged static at 60 °C. Further drying at 80 °C was done to obtain the prepared material in a powder form. The obtained powder was placed in a steel die, and pressed to produce 12.9 mm diameter pellets which were thermally treated at various temperatures (600, 800, and 1200 °C) for 2 h. The studied powder samples were labelled as S1, the as prepared powder, S6, S8 and S12, the samples thermally treated at 600, 800 and 1200 °C, respectively.

### 2.2. Preparation of HAp Coatings on Ti6Al4V Substrates

Ti6Al4V disks of 20 mm of diameter and 4 mm of thickness were polished using different silicon carbide grit up to 1200 grade. The substrates were ultrasonically degreased with acetone for 10 min and washed with distilled water. Finally, the substrates were dried at 200 °C for one hour in an air oven to form a titanium oxide layer. The formation of TiO_2_ layer might decrease the stress concentration and thermal expansion coefficient mismatch between the coatings and the titanium substrate.

These substrates were dip coated in the HAp sol solution, with a dipping and withdraw speed of 12 cm/min. The sol-coated substrates were then immediately transferred into an air oven and held at 80 °C for 30 min to stabilize the deposited layer. To increase the coating thickness, the above process was repeated 3 times and finally it was thermally treated at various temperatures (600, 800 °C) for 2 h. The studied HAp-coating/Ti6Al4V systems are named: C6-HAp/Ti6Al4V for the system thermally treated at 600 °C for 2 h and C8-HAp/Ti6Al4V for the system thermally treated at 800 °C for 2 h.

### 2.3. Characterization of HAp Powder Samples

#### 2.3.1. Thermal Behaviour (DTA/TGA)

The thermal behaviour of the as-prepared powder was carried out using (SETARAM DTA-TG Setsys Evolution-1750, SETARAM Inc., Hillsborough, NJ, USA), with α-Al_2_O_3_ powder as a reference material. A powder sample with a weight of about 50 mg was analysed. The sample and the reference material were heated from ambient temperature to 1300 °C in an argon atmosphere at a heating rate of 20 °C/min.

#### 2.3.2. Fourier Transforms Infrared Spectrometer (FTIR)

The as-prepared sample “S1” and thermally treated samples “S6, S8, S12” were tested by FTIR. Each sample was prepared according to standard procedure by mixing about 2.00 mg of powder sample with 200 mg of KBr, which was subsequently pressed into pellet in an evacuated die. All the spectra were measured by using a Nicolet Magna 550 infrared spectrometer (Nicolet Instrument Corporation, Danbury, CT, USA) which covers the wave number range of 4000–400 cm^−1^.

#### 2.3.3. X-ray Diffraction Analysis (XRD)

X-ray diffraction patterns have been recorded using a Siemens diffractometer D5000 (Siemens AG, Munich, Germany), equipped with scanning rate 0.1 s/step in the 2θ range 10°–100°. The as prepared powder S1 and thermally treated samples; S6 (treated at 600 °C/2 h), S8 (treated at 800 °C/2 h) and S12 (treated at 1200 °C/2 h), were examined with a D8 Discover-Advanced X-ray Diffraction System from Bruker ( Billerica, MA, USA) using monochromatic Cu Kα radiation (λ = 0.15406 nm). The results have been used to study the change of developing phases, crystal structure and degree of crystallinity, with the variation of thermal treatment temperatures.

### 2.4. Characterization of the HAp Sol-Gel Coatings

#### 2.4.1. Coating Thickness

The thickness of the films deposited onto Ti6Al4V substrates was measured by using a stylus profiler (Dektak 6M, Veeco Instruments Inc., Tucson, AZ, USA) and a scanning electron microscope JEOL 6500F (Akishima, Tokyo, Japan). The profiler mechanical method requires the presence of a groove or a step between the substrate surface and the film surface such that the stylus is vertically displaced as it traverses the sample. In our case the coating thickness was measured on half coated samples. A linear scanning (length of 1 mm) was applied for measuring the difference or step produced between the uncoated and coated areas on surface of the sample. For statistical analysis three different areas of each film were studied and the results derived from coating thickness were expressed as mean values ± standard deviation. Cross sectional SEM images were also analysed for estimating the thickness of the samples for contrasting results obtained from both techniques.

#### 2.4.2. Adhesion Measurements

The adhesion tests were performed according to ASTM F1044-05 Standard Test Method. This standard defines the conditions for shear testing of continuous calcium phosphate coatings and metallic coatings adhering to dense metal substrates at ambient temperatures. The method indicates, in a comparative way, the degree of adhesion of the HAp coating to the Ti6Al4V substrate. The test piece consists of two plates, one coated and the other not, joined by an adhesive agent in film form which must have a shear strength of more than 34.5 MPa (5000 Psi). The shear stress is applied parallel to the surface plane of the coating by using an Instron universal test machine. The tests were performed by quintuple.

#### 2.4.3. Roughness Measurements

The roughness of the Ti6Al4V substrate and HAp coatings was evaluated with an Optical Imaging Profiler Sensofar PLμ 2300 (Sensofar-Tech SL, Terrassa, Barcelona, Spain) assisted by standard Dektak 6M software (version 8.35, Veeco Instruments Inc., Tucson, AZ, USA), operating in confocal mode. Measurements were realized by using a confocal objective EPI 20X at a working distance of 4.5 mm with a numerical aperture of 0.45, which results in a spatial sampling of 0.81 μm and a rms resolution of <20 nm. Three different areas were selected on each sample to perform the measurements. On each area, eight roughness determinations were made to obtain the Ra parameter, among others. Concretely, Ra was obtained from a line scanning of 80 μm. The Ra values here included were the medium value obtained as result of the 24 measurements performed on each sample.

### 2.5. Evaluation of the Bioactivity

#### 2.5.1. Immersion Tests of HAp-Coatings/Ti6Al4V System in SBF

The in vitro bioactivity of the prepared HAp-coatings/Ti6Al4V samples was tested by soaking in Kokubo’s simulated body fluid (SBF), which was carried out following the procedure reported in the ISO standard 23317 (Implants for surgery-In vitro evaluation for apatite-forming ability of implant materials). The pH of the SBF solution was buffered at 7.4. The specimens were immersed in separate plastic containers then incubated for 15 days at 37.5 °C. After 15 days the specimens were removed from the solution, rinsed with distilled water and left to dry at room temperature.

#### 2.5.2. Inductively Coupled Plasma Technique (ICP)

Inductively Coupled Plasma technique (ICP) was carried out to determine the Ca^2+^ and PO_4_^3−^ concentration with a PerkinElmer Optima 2100 DV (PerkinElmer Inc., Wellesley, MA, USA). These ions are an indicative of the dissolution and precipitation of apatite from SBF upon the surface of the sample. The concentration was measured as a function of immersion time using ICP. A 20 mL sample of SBF solution was taken from each test tube after 5, 10 and 15 days of immersion and was stored frozen until being analysed by ICP technique. Each of these aliquots was analysed in triplicate.

#### 2.5.3. Scanning Electron Microscopy (SEM)

Studies of the surface morphology of selected specimens, before and after immersion in SBF, were carried out by SEM with a Field Emission Gun Scanning Electron Microscope JEOL 6500F (Akishima, Tokyo, Japan) coupled with an energy dispersive X-ray (EDX) system (Oxford Inca microanalysis system and a windowless detector) for chemical analysis. The samples were examined at a 15 kV or 20 kV accelerating voltage, according to the surface nature. The samples were previously coated with ultra-thin carbon conductive layer to avoid the charging due to the nonconductive nature of the studied samples.

### 2.6. Biocompatibility Studies

#### 2.6.1. Tests with Cell Line of Human Fetal Osteoblasts

The test samples used for the biocompatibility studies were Ti6Al4V disks of 15 mm of diameter, coated with hydroxyapatite obtained via sol-gel and crystallized by applying thermally treated at 600 °C for 2 h. As negative control, for all test samples coverslips Thermanox^®^ (Nunc™ or TMX) of 15 mm of diameter (Thermo Fisher Scientific, Waltham, MA, USA) were used. The test was performed in quadruplicate.

Biocompatibility testing was performed using a cell line of human fetal osteoblasts, subcultures 4-6 (HOb; Health Protection Agency Culture Collections-HPAC-406-05f). A primary right culture was used for this test. The maintenance of this cell line was carried out by performing the passes before reaching 80%–90% of confluence, after a period between 4 and 5 days after seeding, using a trypsin solution (0.5 g/L) and ethylenediaminetetraacetic acid (EDTA; 0.2 g/L) in Hank’s balanced salt solution [20], to release the intercellular junctions and those of the cells with the culture surface.

#### 2.6.2. Cytotoxicity of Leachates. MTT Assay

The MTT assay is a colorimetric assay based on the enzymatic reduction of the tetrazolium dye MTT (3-(4,5-Dimethylthiazol-2-yl)-2,5-Diphenyltetrazolium Bromide) [21,22]. The purpose of applying the MTT assay to this study was to quantify the cytotoxicity of the products released. Firstly, the eluents were obtained from the study materials in a complete culture medium without serum for 1, 3 and 7 days at 37 °C and stirring in sufficient volume to cover the samples. After this step the cells were seeded on 96 well plates (100 μL of packed cells/well) and incubated for 24 h at 37 °C in an atmosphere of 5% CO_2_ using fresh complete culture medium at a concentration of 10^5^ cells/mL and then exchange the culture medium by the leachate from the materials, previously collected at 1, 3 and 7 days and incubating for 24 h at 37 °C in CO_2_ atmosphere. After the steps above described the contents of the wells were removed, instead adding MTT reagent and incubated for 4 h at 37 °C in an atmosphere with 5% CO_2_. After this time the contents of the wells were removed by the addition 100 μL of dimethyl sulfoxide (DMSO) into each individual well.

The reagent MTT binds enzymatically to mitochondria of living cells to form a product blue/purple. The colour intensity produced is related to the number of viable cells, and thus, directly to their proliferation in vitro. In order to quantify the cell viability absorbance measurements were performed at an optical density 570 nm by using a spectrophotometer BioTek ELx808UI (BioTek Instruments Inc., Winooski, VT, USA), with a reference wavelength of 630 nm.

#### 2.6.3. Analysis of Cell Adhesion and Proliferation on the Surface of the Materials. Alamar Blue™ Assay

The temporal evolution of cell proliferation on the HAp coatings was directly assayed in cultures, over a period of 14 days using the reagent Alamar Blue™ [23,24]. This assay allows to perform various measures of cell activity over a period of time, since the reagent used is not toxic to cells cultured in contact therewith. Cellular activity results in the reduction of the reagent added and produces colour changes, which can be measured colorimetrically.

First, the seeding of human fetal osteoblasts, subcultures 4 to 6 (HOb; Health Protection Agency Culture Collections HPAC-406-05f), at a concentration of 10^5^ cells/mL in fresh culture medium was performed on sterile samples, in 24-well plates (1 mL cell concentrate/well). After incubating these plates for 24 h at 37 °C under CO_2_ atmosphere, the culture medium was removed, the samples were washed with PBS, adding in place a 10% solution of the Alamar Blue reagent in complete medium free of phenol red (1 mL/well), which has been maintained in contact with the cultures for 4 h at 37 °C in a CO_2_ atmosphere. After that, the contents of the wells were transferred to 96 well plates (100 μL/well; *n* = 16), replenishing with fresh complete medium in the initially seeded plates (1 mL/well). Optical density reading was performed on a BioTek ELx808IU spectrophotometer (BioTek Instruments Inc., Winooski, VT, USA) at 570 nm, with a reference wavelength of 630 nm, of the samples, subtracting the absorbance values measured for the blank at the values obtained for the different samples.

The protocol described above was repeated at 3, 7 and 14 days, obtaining the pattern that models the proliferation of osteoblasts on the surface of TMX and the coatings of HAp on Ti6Al4V, during the 14 days that the test lasted.

#### 2.6.4. Analysis by SEM of the Cultures Established on the Materials Surface 

The analyses by SEM of the cultures established on the materials surface were carried out on sterilized Ti6Al4V samples coated with crystalline sol-gel-derived HAp and control TMX respectively. Cells of human fetal osteoblasts, subcultures 4 to 6 (HOb; Health Protection Agency Culture Collections HPAC-406-05f) (*n* = 2) in fresh medium with full culture (10^5^ cells/mL) were seeded in 24 well plates (1 mL of concentrated cells/well), and incubated for 24 and 48 h at 37 °C in an atmosphere of CO_2_.

Subsequently, the culture medium was extracted by adding 1 mL per well of a dissolution to 2.5% solution of glutaraldehyde in 0.1 M sodium cacodylate buffer, remaining 4 h in darkness at room temperature. Then the samples were washed with sterile distilled water and dried at 37 °C. Finally, the samples were metalized with chromium and observed under SEM by using a Hitachi SU8000 (Hitachi Ltd., Chiyoda-ku, Tokyo, Japan).

### 2.7. Corrosion Protection Behaviour

The corrosion protection behaviour of the crystalline sol-gel-derived Hap coatings deposited on Ti6Al4V surfaces was evaluated by electrochemical impedance spectroscopy (EIS). These electrochemical measurements were performed with an Autolab potentiostat/galvanostat PGSTAT302N equipped with a FRA32M frequency response analyzer module (Metrohm-Autolab, Utrecht, The Netherlands). A standard three-electrode cell was used for this purpose. The working electrode was the studied sample with an area of 3.14 cm^2^. The reference and the counter-electrode were a saturated calomel electrode (SCE) and a large size graphite sheet, respectively. The electrochemical cell was filled with Kokubo’s simulated body fluid. The EIS measurements were made at open circuit potential (OCP). Logarithmic frequency scans were carried out by applying sinusoidal wave perturbations of ±10 mV in amplitude, in the range of 10^5^–10^−3^ Hz. Five impedance sampling points were registered per frequency decade. The impedance data were analysed by using the ZView^®^ software, version 3.5b (Scribner Associates Inc., Southern Pines, NC, USA).

## 3. Results and Discussion

### 3.1. Characterization of HAp Powder Samples

**Figure 1:**
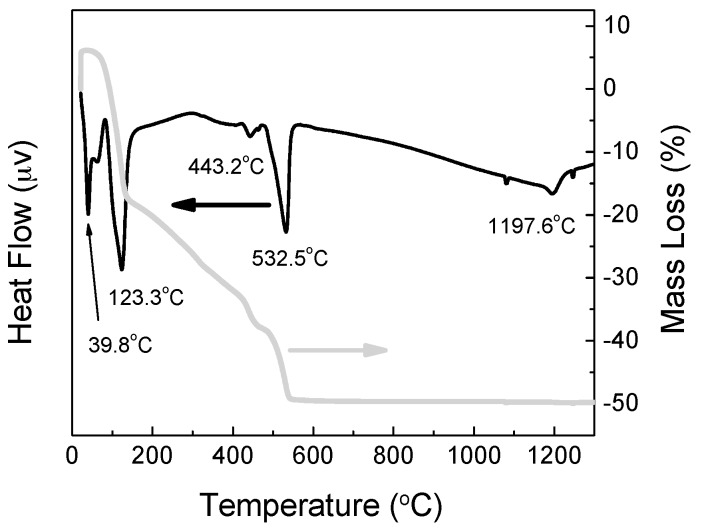
DTA/TG of the as prepared powders.

Differential Thermal Analysis (DTA) and thermogravimetry (TG) plots of the as-prepared powder are shown in Figure 1. The DTA was employed to determine the temperature at which the as-prepared powder, amorphous calcium phosphate (ACP), transforms to crystalline HAp.

In the DTA curve of the dry gel (powder), the first endothermic peak appeared at 38.8 °C is due to the evaporation of the absorbed water (residual moisture evaporation) [25]. The two endothermic peaks at 123.3 °C and 443.2 °C are attributed to elimination of crystalline water in un-reacted calcium nitrate tetra hydrate Ca(NO_3_)_2_·4H_2_O and removal of NO^3−^ groups, as well as, to the condensation “dehydration” of hydrogen phosphate ions (HPO_4_^2−^) to form amorphous pyrophosphates (P_2_O_7_^4−^), which are a component of the amorphous calcium phosphates that are formed during the processing of HAp synthesis [26,27,28]. The endothermic peak at 532.5 °C is representative of the endothermic reaction indicating the crystallization of HAp. The last endothermic peak observed at 1197.6 °C may be attributed to the decomposition of the HAp [29].

In the TG, the weight loss was complete at about 570 °C and three discrete weight-loss regions occurred as seen in Figure 1. The first region at 44–172 °C with 25% weight loss corresponds to the elimination of adsorbed water. This process was indicated as endothermic peak at 38.8 °C and shoulder at 60 °C in the DTA diagram. The second weight loss region appear at 172–479 °C in the TG plot with a 18% weight loss which corresponds to the elimination of lattice water and was indicated as endothermic peak in the DTA diagram at 443 °C. The third weight loss observed in the TG diagram at 479–570 °C with a weight loss of 11% corresponds to the decomposition of the nitrate salts which is also indicated as an endothermic peak at 532 °C in the DTA curve.

**Figure 2:**
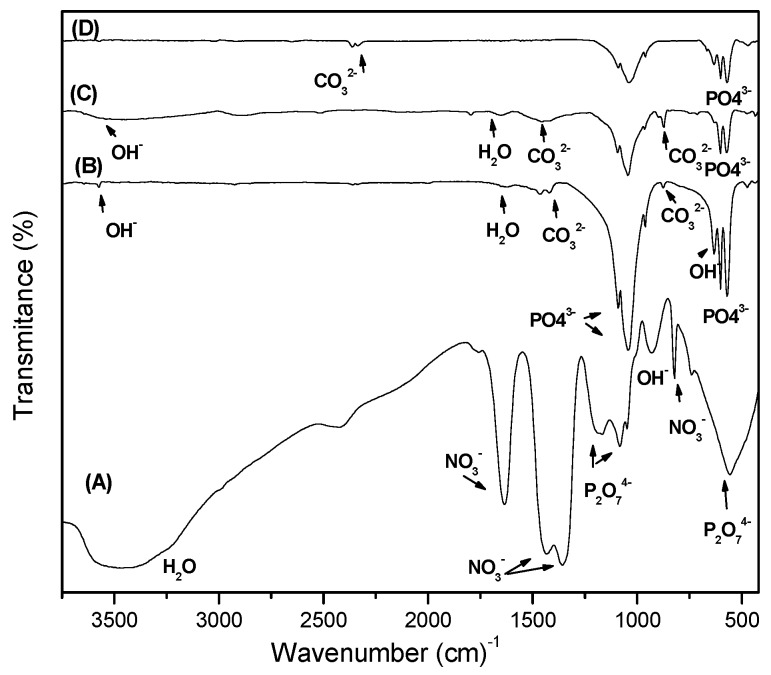
FTIR spectra of as-prepared powders (**A**) and thermally treated at different temperatures; 600 °C/2 h (**B**), 800 °C/2 h (**C**) and 1200 °C/2 h (**D**).

Fourier transform infrared spectroscopy (FTIR) is an analytical technique widely used to characterise the functional groups within the structure of biomaterials such as HAp, which is IR active. The FTIR spectra of the as-prepared sample S1 and thermally treated samples S6, S8 and S12 are shown in Figure 2.

The main peaks observed are situated in the region of 900–1200 cm^−1^ which are due to the presence of P-O groups of HA. These peaks are present in all thermally treated samples. Theoretically, there are four vibrational modes present for phosphate ion ν_1_ (961 cm^−1^), ν_2_ (475–440 cm^−1^), ν_3_ (1190–976 cm^−1^) and ν_4_ (660–520 cm^−1^), all the four modes are IR active and are used to characterize apatite structure. 

The FTIR spectrum of the as prepared sample shows a very poorly defined broadband in the region where bands due to phosphate ions in the HAp should be present (spectrum A in Figure 2). In spectra of S1, the intense broad peak at 1342 cm^−1^ and the sharp peaks at 825 cm^−1^ and 1631 cm^−1^ along with the small peaks of 1049 and 1766 cm^−1^ are related to NO_3_^−^ bonds which come from the starting precursor. As the temperature increases, NO_3_^−^ could decompose into gaseous oxide of nitrogen and, hence, the related absorption bands disappear as the temperature increase [30]. The peaks at 740, 1083, 1164 and 555 cm^−1^ indicate the presence of P_2_O_7_^4−^ in the as prepared sample dried at 80 °C. These peaks also disappeared as temperature increased [30]. The broad band at 3435 cm^−1^ and the band at 914 cm^−1^ assigned to OH^−^ stretching and bending of the lattice water both of which are weakened, as the powder was heated at 600 °C/2 h. All of these mentioned bands are indicatives of poorly crystalline or amorphous apatite structure.

The spectra of the S6, S8 and S12 samples thermally treated at 600, 800, 1200 °C/2 h, respectively exhibited a decrease in the intensity of OH^−^ peaks and disappearance of NO_3_^−^ and P_2_O_7_^4−^ bands indicating the transformation of amorphous HAp into crystalline form. This is confirmed by the appearance of new absorption bands at 520–1500 cm^−1^ assigned to PO_4_^3−^ groups, which are presents in the HAp.

The incorporation of carbonate is a common phenomenon during the formation of biological apatite in the human body. In the synthesized HAp, the carbonate substitution may result from CO_2_ in the atmosphere, dissolved in the solutions used for the preparation of the HAp particles. The CO_3_^2−^ can easily replace OH^−^ (A sites) and PO_4_^3−^ (B sites) at 1500–1545 cm^−1^ and 1420–1470 cm^−1^, respectively to form carbonated HAp [30].

In the spectrum of the 600 °C/2 h thermally treated sample S6 the two weak peaks at 1465, 1419 cm^−1^ (ν_3_) and a small shoulder observed at 875 cm^−1^ indicate that the CO_3_^2−^ groups were substituted for PO_4_^−3^ groups, forming B-type carbonated apatite [31] (spectrum B in Figure 2). These carbonate bands appeared in the 800 °C/2 h thermally treated sample S8 at 1466 and 870 cm^−1^ (spectrum C in Figure 2).

In the Spectrum of the 1200 °C thermally treated sample S12 the new carbonate bands which appear at 2320–2348 cm^−1^ (combination of ν_1_ + ν_3_), 1467–1412 and 878 cm^−1^ are observed (spectrum D in Figure 2). The decomposition of CaCO_3_ is well known to occur at 580 °C or higher temperatures, so the CO_3_^2−^ bands were expected to be absent in the spectra of the S6, S8 and S12 samples. However, the presence of the carbonate peaks is still evident in these spectra, which further proves the inclusion of CO_3_^2−^ groups in the apatite structure. From the biological point of view, the presence of a small amount of carbonate in the HAp structure is an advantage and has similarity to the natural bone mineral which contain 4%–6% carbonate by weight [31]. The carbonated HAp (CHAp) exhibit higher bioactivity than that of HAp. This is attributed to be due to the greater solubility of CHAp.

According to Landi et al., the fraction of crystalline phase (*X*_C_) can be calculated through the equation below [32]:

XC=1−V112300I300

*I*_300_: Intensity of peak diffracted from the (300) crystallographic planes of HAp.
*V*_112/300_: Intensity of the valley between the peaks of the planes (112) and (300).

The average crystallite size was calculated from the broadening in the XRD pattern. According to the Scherrer’s equation [33]:

$$L_{c}=\frac{K\lambda }{\beta \cdot \cos\theta }$$

*L*_c_: Average crystallite size (nm).
*K*: Shape coefficient (value between 0.9 and 1.0).
λ: Wavelength of X-ray beam-Cu Kα radiation (λ = 0.15406 nm).
β: Full width at half maximum (FWHM) of HAp(211).
θ: Diffraction angle.

The diffraction peak corresponding to the (002) was chosen for calculation of the crystalline size, as it is relatively sharper than the other peaks.

**Figure 3:**
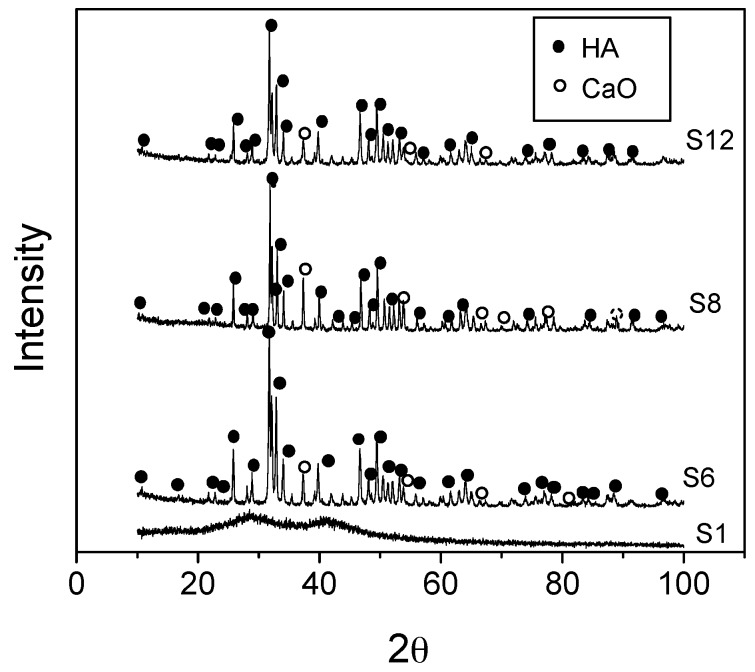
XRD patterns and developed phases of the dried gel (S1) and the powders thermally treated at different temperatures; 600 °C/2 h (S6), 800 °C/2 h (S8) and 1200 °C/2 h (S12).

Figure 3 illustrates the XRD patterns of S1 aged gel, dried at 80 °C and of the S6, S8, and S12 samples respectively. One major peak appears around 31.81 2θ for S6, S8 and S12 samples, represent the characteristic peak of HAp apatite phase (JCPDS09-0432).

The XRD pattern of the as-prepared sample S1 is mainly amorphous, and there is no obvious diffraction peak observed (Figure 3). In the case of the thermally treated samples, S6, S8 and S12, the increase of the temperature of the thermal treatment produces significant changes in the crystalline structure of the samples. These changes are reflected in the appearance of several peaks, which are ascribed to the diffraction peaks of the HAp phase. The XRD pattern of the thermally treated S6, S8 and S12 indicates the existence of calcium oxide as traces, together with HAp as main phase.

The presence of CaO is due to the remaining Ca(NO_3_)_2_, that can be directly transferred to CaO at high temperature [10,34]. The degree of crystallinity and calculated crystal size of the thermally treated samples can be seen in Table 1. The degree of crystallinity of HAp increase as the temperature does from 600 to 800 °C (Table 1), but then decrease for the sample treated at 1200 °C for 2 h. The decrease in crystallinity of the 1200 °C thermally treated sample (S12) is attributed to the decomposition of HAp at this temperature [29]. For the samples S6 and S8, the increase of the crystal size is attributed to an increase of ion migrations favoured when the temperature is increased (Table 1).

### 3.2. Characterization of the HAp Sol-Gel Coatings

**Figure 4:**
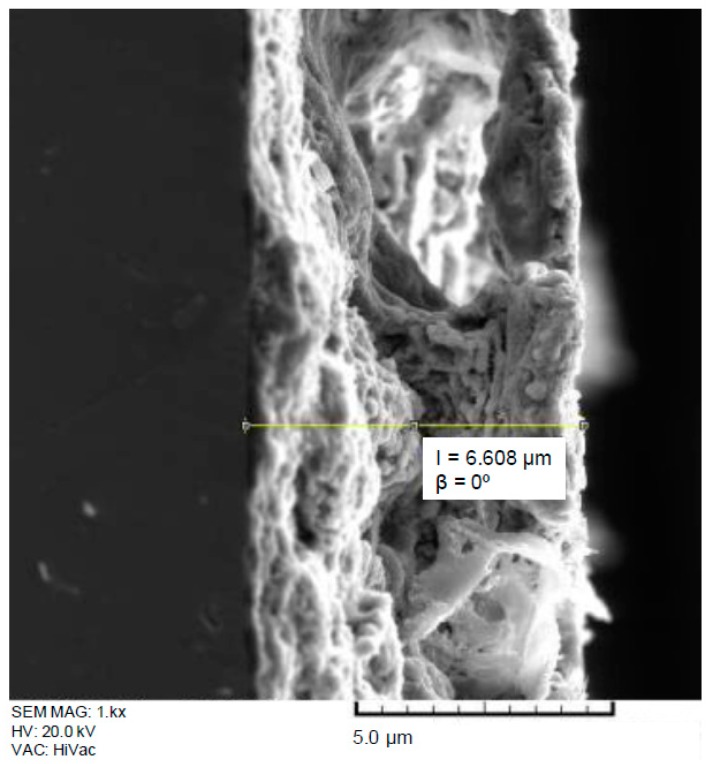
Cross sectional SEM image of a S6 coatings deposited on Ti6Al4V alloy.

Table 2 shows the thickness values of the films deposited onto Ti6Al4V substrates measured with a stylus profiler. These results present a good correlation with those obtained by estimating the coating thickness from cross sectional SEM images (Figure 4). To enhance the barrier properties of the coating it would be interesting to control the treatment parameters in order to increase the thickness of the resulting coatings. Nevertheless, it has been observed that the thicker coatings (i.e., those obtained for the high temperature process of 1200 °C) were more susceptible to the developments of cracks and lack of uniformity, which could be harmful from the point of view of the corrosion protection, if these cracks reach the metal substrate.

Table 3 shows the results obtained by applying the adhesion tests, according to ASTM F1044-05 Standard Method, for the five specimens of the samples S6 based on the HAp sol-gel-derived coatings deposited on Ti6Al4V alloy thermally treated at 600 °C during 2 h. The result of 29.0 MPa is consistent with that reported the ASTM standard for Ti6Al4V alloy coated with HAp, which raises the mean cohesive strength of 29.2 ± 4.8 MPa. 

**Figure 5:**
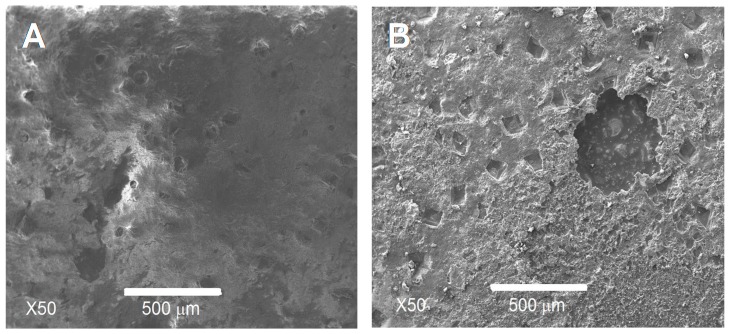
SEM images of the surface resulting from the adhesion test for samples with and without coating: (**A**) HAp coated sample and (**B**) uncoated sample.

**Figure 6:**
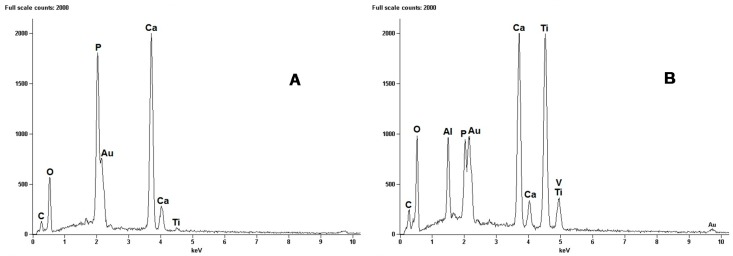
EDX for samples observed by SEM: (**A**) HAp coated sample and (**B**) uncoated sample.

To determine where the break takes place, the surfaces of samples used in the adhesion tests were observed by SEM (Figure 5). It was readily appreciated that the mechanical failure always occurred inside the HAp coating layer and only in a few points between the coating and the adhesive that joins both samples for the test. From these results, it was ensured that the resistance between the HAp coating and Ti6Al4V alloy substrate can reach values higher than those obtained. In order to confirm the above exposed, a global EDX analysis was made on each side of the samples used in the adhesion test, which are shown in the micrographs of Figure 6.

EDX results showed the presence of HAp covering each side of the analysed samples after the adhesion test. These results allow affirming that the rupture occurs inside the coating layer in almost all the tested area and not at the coating/metallic substrate interface. This occurs due to the strong adhesion of the HAp layer onto the surface of the Ti6Al4V alloy. This high adhesion can be a result of a primary chemical bond, produced by the formation of Ca_3_(PO_4_)_2_ and CaTi_2_O_5_, generated during the thermal treatment of crystallization of HAp films. From these results we can say that the HAp coatings obtained by the sol-gel methodology here proposed, have enough adhesion to be used as coatings in metallic orthopaedic prosthesis.

**Figure 7:**
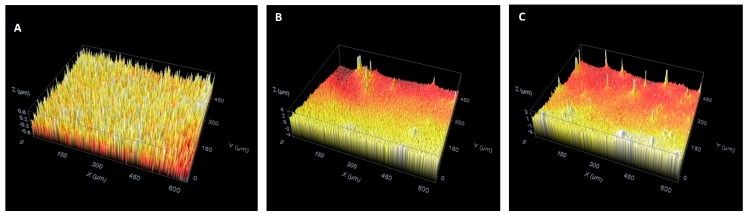
Confocal microscope three-dimensional images of the uncoated Ti6Al4V surface (**A**) and HAp coatings on Ti6Al4V thermally treated at 600 °C/2 h (**B**) and 800 °C/2 h (**C**), repectively

Confocal microscopy was applied to study the roughness and topography of the surface of the prepared films. Figure 7 shows the topographic images taken of the uncoated Ti6Al4V surface (Figure 7A) and the HAp coatings on Ti6Al4V thermally treated at 600 °C/2 h (Figure 7B) and 800 °C/2 h (Figure 7C), respectively.

The roughness of the film is a critical factor that affects the implant fixation period and the fixation strength with the body tissue. The roughness parameters that were obtained from 3D image analysis by means of confocal microscopy technique are summarized in Table 4.

The Ra roughness value increases for the HAp coated samples thermally treated at 600 °C/2 h and 800 °C/2 h when compared to the uncoated Ti6Al4V sample (Table 4). This could be due to the evaporation of solvents during thermal treatment.

### 3.3. Bioactivity Assessments of the HAp-Coatings/Ti6Al4V System

**Figure 8:**
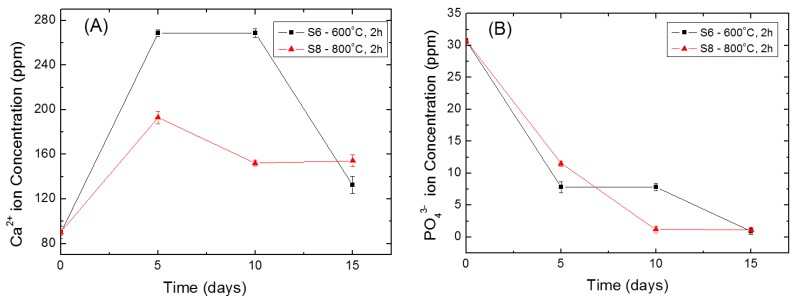
Variations of the (**A**) Ca^2+^ and (**B**) PO_4_^3−^ concentrations in SBF during immersion test.

The bioactivity of artificial materials can be attributed to the formation of biologically active bone-like apatite layer. This layer can be formed onto the surface of the sample upon immersion in Kokubo’s simulated body fluid [35,36,37]. The concentration of Ca^2+^ and PO_4_^3−^ in SBF after immersion of the prepared samples is shown in Figure 8.

An increase of the Ca^2+^ concentration accompanied by a decrease of PO_4_^3−^ in SBF is observed during the first 5 days (Figure 8A,B). When the immersion time exceeds 5 days, the concentration of both ions decreases continuously. The increase in the concentration of Ca^2+^ ions is due to its release from the samples into the SBF solution. This will accelerate the nucleation of apatite by increasing the ionic activity products of the apatite, i.e., make the solution more saturated with respect to the sample immersed in it. Once the apatite nuclei are formed, it will grow continuously by consuming the Ca^2+^ and PO_4_^3−^ ions from the solution. Thus, the concentration of both SBF components decreases with the time. This indicates that the HAp-coatings/Ti6Al4V thermally treated at 600 and 800 °C/2 h are bioactive.

**Figure 9:**
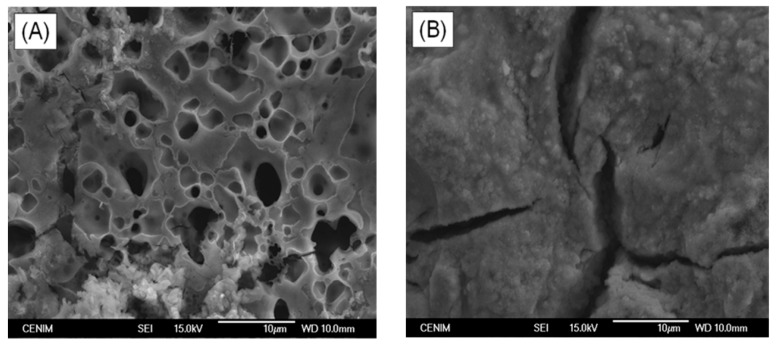
SEM micrographs obtained for the HAp-coatings/Ti6Al4V system thermally treated at (**A**) 600 °C/2 h; (**B**) 800 °C/2 h before immersion in SBF.

**Figure 10:**
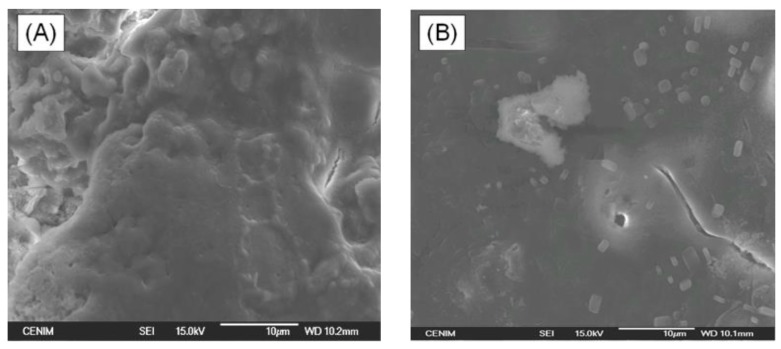
SEM micrographs obtained for the HAp-coatings/Ti6Al4V system thermally treated at (**A**) 600 °C/2 h; (**B**) 800 °C/2 h after immersion in a SBF solution.

Studies of the surface morphology of selected specimens, before and after immersion in SBF, were carried out by SEM. SEM micrographs obtained for the HAp-coatings/Ti6Al4V system thermally treated at (A) 600 °C/2 h; (B) 800 °C/2 h before immersion in SBF are shown in Figure 9. After 15 days of immersion in SBF the surface of both samples is covered with a bone-like apatite layer which proved that both samples are bioactive. The surface of the sample thermally treated at 600 °C/2 h is completely covered with these precipitations from SBF (Figure 10A), while the surface of the sample thermally treated at 800 °C/2 h is not completely covered, as still some cracks are observed by SEM at the surface (Figure 10B). Nevertheless, these cracks do not reach the metal surface. In this way, the HAp coating is able to act as a barrier against the aggressive SBF solution and to protect the Ti6Al4V alloy against corrosion.

### 3.4. Biocompatibility Evaluation of Sol-Gel-Derived HAp Coatings Deposited on Ti6Al4V Surfaces. Cell Viability and Proliferation

#### 3.4.1. Cell Viability-Absorbance Measurements

The cell viability and cytotoxicity of the products released was quantified by using MTT assays [21,22]. For such a purpose absorbance measurements were carried out with a Biotek ELX808IU detector using a test wavelength of 570 nm and a reference wavelength of 630 nm.

The absorbance values are subtracted from the measured values for the blank target and are relativized with respect to the values read for the TMX control, obtaining the relative cell viability (% *VR*) with respect to this control, from the equation:

$$\%VR=\frac{OD_{S}-OD_{B}}{OD_{C}-OD_{B}}\times 100$$

where, *OD*_S_, *OD*_B_ and *OD*_C_ are the measures of optical density of the sample, blank (MTT reagent solution in culture medium without cells introduced into wells) and negative control (TMX), respectively.

**Figure 11:**
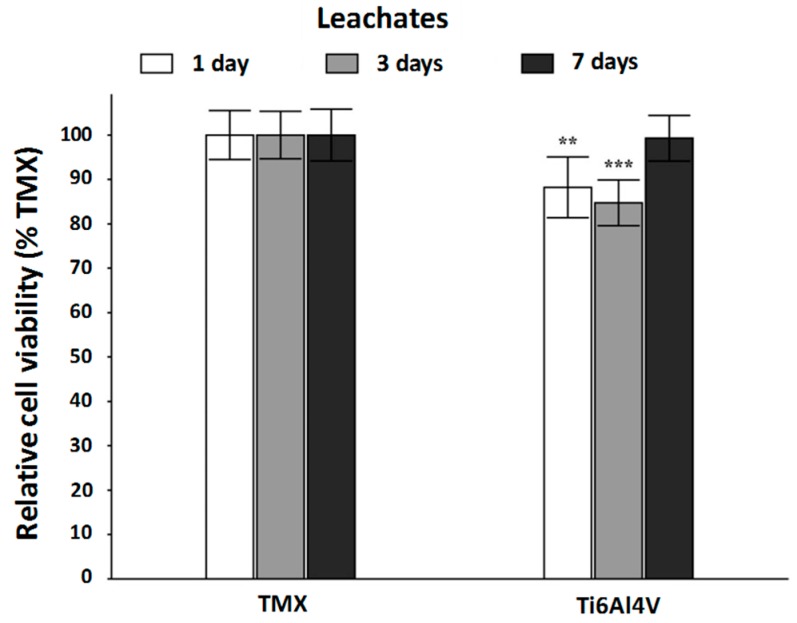
Results of the MTT assay test for human osteoblast cultures maintained with leachate at 1, 3 and 7 days obtained from samples of the control TMX and study materials (** *p* < 0.01; *** *p* < 0.001).

The results obtained from the MTT assay are represented in Figure 11. This figure shows the average value of the relative cell viability ± confidence interval of 95% (*n* = 8) and the significance level obtained in the analysis of variance compared against the values obtained for TMX.

As shown in Figure 11, the leachates obtained after 1 and 3 days have a measureable influence on the viability of the cells maintained in culture contact them for 24 h. Nevertheless, the decrease in the relative cell viability with respect to the samples of the negative control is not too accused, being in both cases these viability values above 80% of the measurement for that control. In contrast, the leachate obtained at 7 days does not significantly affect the viability of the osteoblasts used as a model, being measured in this case 99.30% ± 5.15% cell viability. It can be concluded, that the leachate obtained during a period of 7 days, from samples of the experimental system studied, did not show a significant toxicity for the cultures of the osteoblasts used as model.

#### 3.4.2. Cell Proliferation-Alamar Blue™ Assay

The method used for measuring cytotoxicity and cellular proliferation on the surface of the materials was the Alamar Blue™ test [23,24]. The results obtained in the Alamar Blue™ assay showed firstly, that between 1 and 3 days after seeding, the cellular activity of the cultures established on the sol-gel-derived HAp coatings underwent an initial decrease with respect to those set on the samples of the negative control. Subsequently, the levels of cellular activity increase while reaching the measured values for the control samples in any case. At all times, the measured values of cellular activity in the experimental system are significantly lower than those measured for the TMX control.

**Figure 12:**
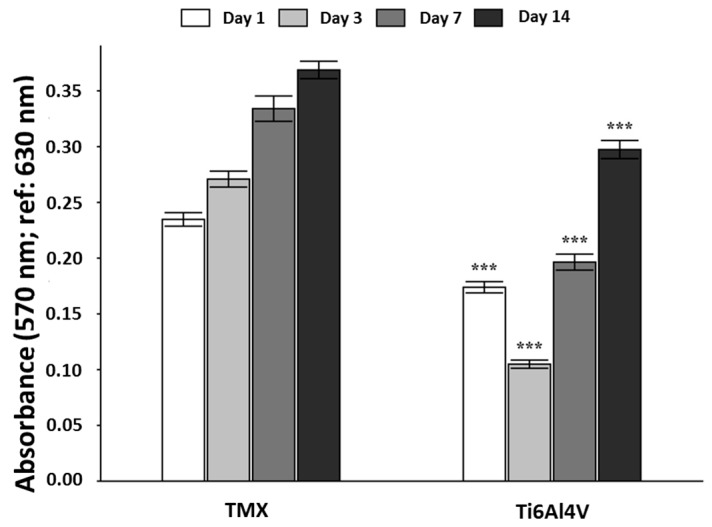
Absorbance measurements obtained after using Alamar Blue™ assay for cultured human osteoblasts seeded on the TMX control and the HAp sol-gel-derived coating/Ti6Al4V system studied (*** *p* < 0.001).

Figure 12 represents the mean ± confidence interval 95% (*n* = 16) and the level of significance was obtained in the analysis of variance carried out by comparing the values obtained for the cultures seeded on the Ti6Al4V samples coating with HAp compared to the values obtained for the cultures seeded on TMX.

As it is observed, the decrease in cellular activity measured after 3 days of culture is accused, caused by the difficulty of a high proportion of cells to adhere initially on the surface of the system. Subsequently, cell activity measured for the cultures seeded on the experimental system increases in both absolute and relative terms, with respect to the measured values for the control samples after 7 and 14 days of culture. In fact, cellular activity reaches values around 60% to 80% with respect to TMX control, after 7 and 14 days of culture, respectively. This observation can be justified by the fact that on the surface of the HAp coatings exists a greater availability of potentially colonisable space by osteoblasts grown, that once correctly attached, can spread at a higher speed than in the control samples, where the availability of space is much lower. Another factor which is crucial to the cell adhesion, that it may be involved in the increase of the cellular activity, is the adsorption of proteins on the surface of HAp, fact that can promote their proliferation after an initial phase of which was not yet met the optimum conditions for the proliferation of the cells used.

#### 3.4.3. Inspection of the Cultures Established on the Materials Surface-SEM

**Figure 13:**
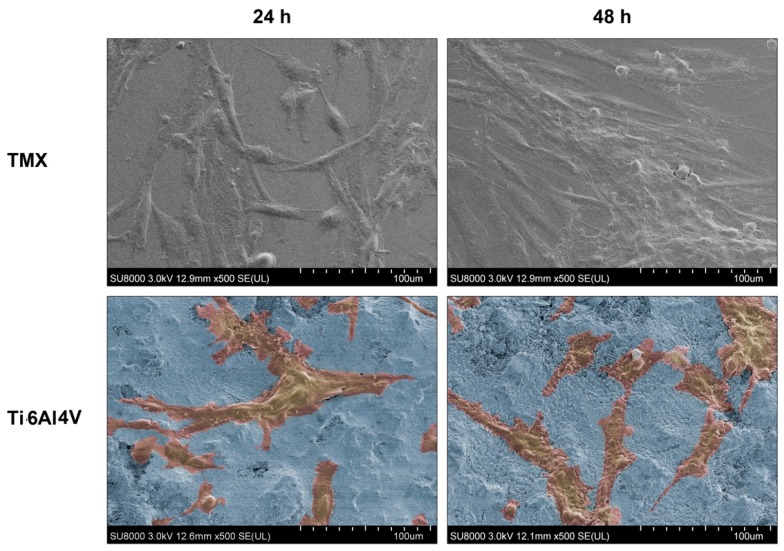
SEM images (×500) of cultures of human osteoblasts seeded on the surface of the TMX control and studied system, after placing samples in culture for 24 and 48 h. A false colour reconstruction has been used to show the proliferation of the human osteoblasts

As shown in Figure 13, on the surface of Ti6Al4V samples coated with HAp, cells with cytoplasmic extensions (filopodia and pseudopodia) appear for 24 and at 48 h of culture. This indicates that both cells are adhered to the substrate, although it is not possible to find differences in the density and state of the cells when comparing the cultures maintained during 24 and 48 h. The results obtained in this test indicate that the evaluated system showed low cytotoxicity and promoted the adhesion and proliferation of human osteoblasts in culture.

### 3.5. Corrosion Behaviour

**Figure 14:**
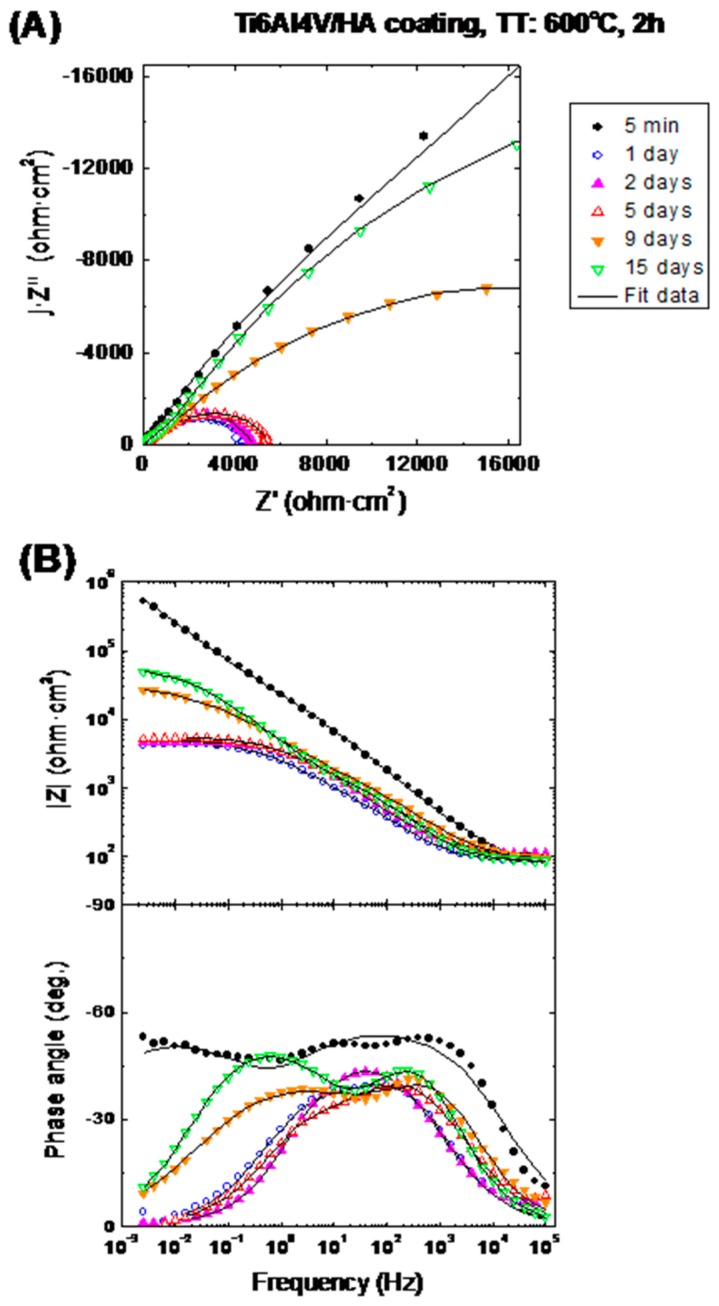
Evolution of the electrochemical impedance spectra with immersion time in SBF for a HAp-coatings/Ti6Al4V system thermally treated at 600 °C, 2 h.

**Figure 15:**
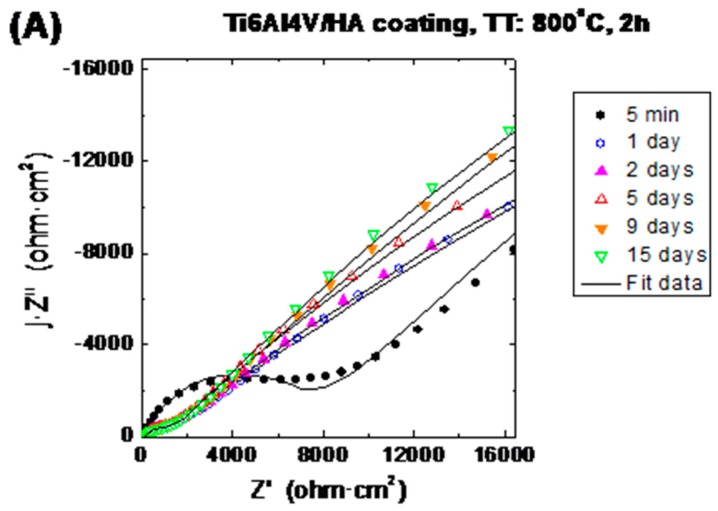

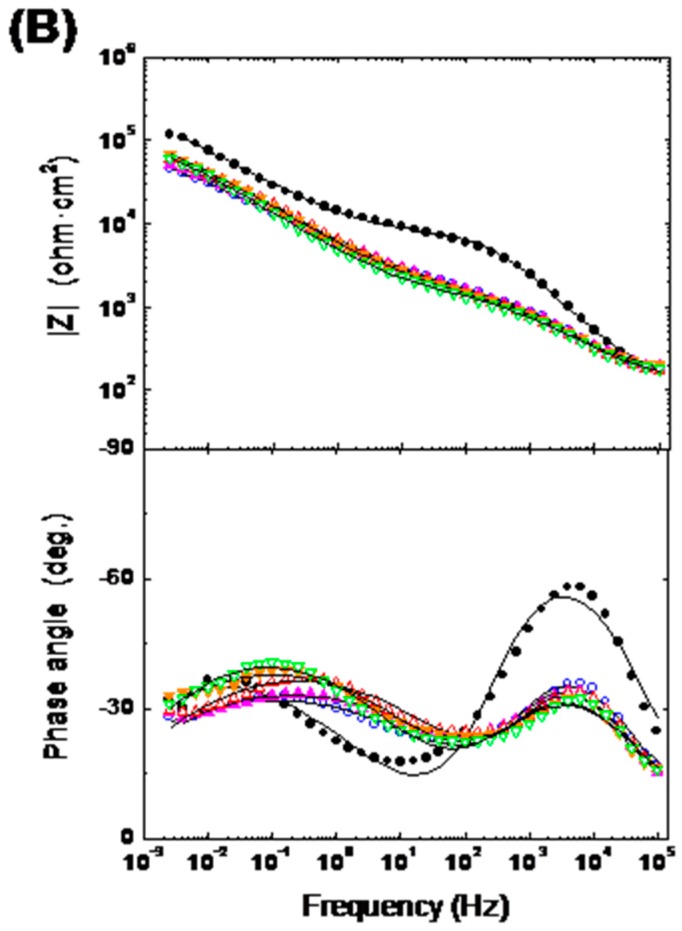
Evolution of the electrochemical impedance spectra with immersion time in SBF for HAp-coatings/Ti6Al4V system thermally treated at 800 °C, 2 h.

Electrochemical impedance spectroscopy (EIS) was used for evaluating the barrier properties of the HAp coatings and the corrosion behaviour of the HAp-coatings/Ti6Al4V systems. Figure 14 and Figure 15 show the evolution of the impedance plots with the immersion time in SBF for the HAp-coatings/Ti6Al4V systems thermally treated at 600 °C/2 h and 800 °C/2 h, respectively. Figure 14A and Figure 15A show the impedance data represented as Nyquist plots, where the imaginary part (Z′′) of the impedance vector **Z**(f) is represented in the complex plane against the real part (Z′) of this vector, with the frequency (f) as implicit variable [38,39]. Figure 14B and Figure 15B show the same impedance data in Bode representation. In the Bode diagrams the logarithm of the magnitude of the impedance vector (log |**Z**|) and the phase angle (θ) are plotted against the logarithm of frequency (log f) of the applied sinusoidal electric voltage signals [39].

Electrochemical techniques are well suited to monitor changes over time of the interface between metal surfaces and aqueous solutions [39,40,41]. From EIS, information on the interface capacitance (thus oxide films, adsorption processes) and the interface resistance (charge transfer processes, dissolution of ions from metal alloys as for instance metallic implant materials) can be obtained [16,40,41]. In addition, in the studies of coated metals, information about the protective nature of the coating can be also obtained [42]. In this case, the coating capacitance provides information on the thickness and the degree of surface coverage [43,44,45]. The coating capacitance value usually changes over the time in contact with aqueous solutions. The change depends on the barrier properties afforded by the coating against the ingress of water [43,44,45]. Finally, the coating resistance provides information about the ionic resistance of coating systems immersed in aqueous solutions. The changes of the ionic resistance with the immersion time are due to the penetration of the electrolyte through the intrinsic porous of the coating [43,44,45]. All these concepts can be extrapolated to the studies related with the corrosion behaviour of coated metallic implants in contact with the simulated body fluids.

#### 3.5.1. Selection of the Electrical Equivalent Circuit

**Figure 16:**
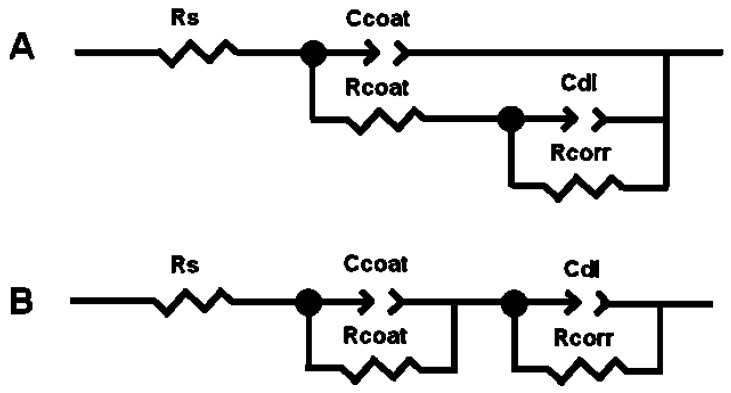
Electrical Equivalent Circuits (EECs) used to analyze the experimental electrochemical impedance data. (**A**) Parallel Maxwell forms; (**B**) Series Voigt forms [38].

An interface undergoing an electrochemical reaction is typically analogous to an electronic circuit consisting of a specific combination of resistors and capacitors. Thus, the electrochemical system can be described in terms of its equivalent circuit. The experimental impedance data can be modelled using complex non-linear least-square (CNLS) fit analyses and suitable electrical equivalent circuits (EECs). Two equivalent circuits have been used for this study (Figure 16).

The EEC showed in Figure 16A has been successfully used in a lot of studies related with the corrosion protection behaviour of organic coatings on metal surfaces [42,43,44,45]. The EEC showed in Figure 16B has been also used several times with providing also successful results [16,42]. Following the notation of the ZView commercial software, in both electrical equivalent circuits *R*_s_ represents the solution resistance of the bulk electrolyte. *C*_coat_ is the coating capacitance. Because of inhomogeneities in the coating, *C*_coat_ has to be implemented as a Constant Phase Element (*CPE*_coat_). *R*_coat_ is the coating pore resistance. *R*_corr_ represents to the corrosion resistance of the metal substrate and *C*_dl_ is the double layer capacitance at the metal/electrolyte interface, both at the base of pores and perforations of the coating. Due to inhomogeneities in the coating and metal surface, this capacitance has also to be implemented as a *CPE*_dl_.

Mathematically, the *CPE*’s impedance is given by the following equation [46]:

*Z*(*CPE*) = 1/[*Y*_0_(*j*·ω)*^n^*]

where *j* = $\sqrt{-1}$, ω is the angular frequency ω = 2π*f*, and *f* is the frequency in Hz. The *CPE* is defined by two parameters, *CPE*-*Y*_0_ and *CPE*-*n*. *CPE*-*Y*_0_ is a fit parameter and the exponential factor *CPE*-*n* is related with a non-uniform current distribution due to the surface roughness or other distributed properties, and varies between 0 and 1. The *CPE* reduces to a resistor for *CPE*-*n* = 0, to a Warburg element representing semi-infinite length diffusion phenomena for *CPE*-*n* = 0.5 and to an ideal capacitor for *CPE*-*n* = 1.

Good results have been obtained by applying the electrical equivalent circuit showed in Figure 16A, to fit and simulate the experimental impedance plots obtained for the two systems studied in the present work, C6-HAp/Ti6Al4V and C8-HAp/Ti6Al4V (Figure 14 and Figure 15). However, simultaneous agreement between calculated and measured impedance spectra does not necessarily mean that the circuit model is a unique representation of the spectra. One cannot assume the uniqueness of a circuit model merely on the basis of a good fit observed experimental spectrum [47].

Table 5 shows an example where the two equivalent circuits showed in Figure 12 provide very similar fit results when they are applied to the C8-HAp/Ti6Al4V system, after 5 days of immersion in SBF.

Table 5 also shows the chi-square (χ^2^) values obtained with both equivalent circuits. The χ^2^ parameter gives an estimation of the quality of the fitting results to the experimental impedance spectra. In all the tested samples the quality of the fitting results was also very good, independently of the two electrical equivalent circuits used for obtaining the fits, because the χ^2^ obtained values were lower than 10^−3^. Nevertheless, the equivalent circuit showed in Figure 16A gives as a result ambiguous values for the *CPE*_dl_-*n* parameter when it is applied to the system thermally treated at 600 °C/2 h. Thus, for 1 and 2 days of immersion in Kokubo solution, the calculated values for *CPE*_dl_-*n* are equal to 9.268 and 1.933 respectively (Table 6). These results are very far from the range of values with a physical meaning (0.5 ≤ *CPE*_dl_-*n* ≤ 1). In the case of the systems thermally treated at 600 °C/2 h, for 1 and 2 days of immersion in Kokubo solution, the relative error obtained for *CPE*_dl_-*n* values when applied to the equivalent circuit is also very bad (Table 6). On the other hand, the equivalent circuit showed in Figure 16B provides very successful fitting results for all the experimental impedance plots obtained in this study. Thus, this equivalent circuit was finally chosen for analysing all the impedance data. 

#### 3.5.2. Interpretation of the Impedance Spectra

Figure 14 and Figure 15 show the evolution of the impedance plots as a function of the immersion time in Kokubo solution of the HAp-coatings/Ti6Al4V systems thermally treated at 600 °C/2 h and 800 °C/2 h. Both figures show also the obtained plots by fitting using the equivalent circuit showed in Figure 16B. Initially, both samples exhibit very similar EIS behaviour. For short immersion time (5 min) the Nyquist plots draw very open arcs (Figure 14A and Figure 15A). At the beginning of the immersion test, both samples show also high impedance modulus for all the tested domain of frequencies, such it can be observed in the Bode representation of the logarithm of the impedance magnitude against the logarithm of the frequency (Figure 14B and Figure 15B). For this short immersion time both figures show that in the high frequencies range, the phase angle presents the highest values. High values of the phase angle at high frequencies are typically associated to barrier effects of coatings, which are due to their dielectric properties.

**Figure 17:**
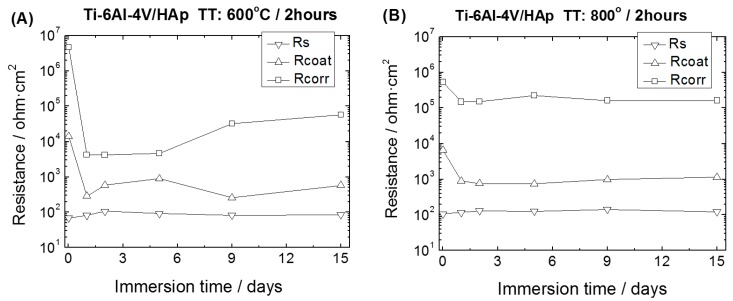
Evolution of the *R*_s_, *R*_coat_ and *R*_corr_ values with immersion time in SBF of HAp-coatings/Ti6Al4V systems. Thermal Treatments: (**A**) 600 °C/2 h and (**B**) 800 °C/2 h.

Figure 17 shows the obtained values of the resistances obtained by using the equivalent circuit showed in Figure 16B. In both cases the *R*_s_ values are similar and remain without significant changes along the immersion time. For the HAp-coatings/Ti6Al4V systems obtained at 600 °C/2 h, the *R*_coat_ values shown a slight decrease due to the electrolyte inwards within the first day of immersion. After the first day, the *R*_coat_ values showed small fluctuations, increasing slightly and then decreasing again after six days of immersion. These small fluctuations are associated with the changes observed in the *R*_corr_ values. In this case, the *R*_corr_ values are sensitive to the changes occurring at the Ti6Al4V alloy/SBF interface at the base of the porous. After the first hours of immersion, the *R*_corr_ value decreases considerably which indicates that the electrolyte reaches the Ti6Al4V surface, in several microareas, and the activity in these microareas is a dominant effect. In these microareas, according to the passivity theory, in aqueous solution have simultaneously both active and passive surfaces and undergo a continuous process of partial dissolution and repassivation.

Depending on the extent of the Ti6Al4Vsurface that is in direct contact with the SBF, this process will be more or less important and will dominate the impedance response. For the sample thermally treated at 600 °C/2 h, this continuous process of partial dissolution and repassivation seems to be dominant along the next 5 days of immersion giving as a result a constant value of 4 × 10^4^ ohm·cm^2^ within this period of time. After that, it is interesting to observe that the values of the *R*_corr_ parameter increase quickly and reach values closer to those observed at the beginning of the immersion tests (Figure 17A). This behaviour can be due to the blocking of the pores by the formation of precipitates from the solution, which prevent further penetrations of the SBF impeding the growth of active microareas at the base of the HAp-coating pores.

For the HAp-coatings/Ti6Al4V systems obtained at 800 °C/2 h, the *R*_coat_ values showed a slight decrease due to the electrolyte inwards within the first day of immersion, attaining then a constant value of *R*_coat_ (Figure 17B), which shows a good stability of the coating along the immersion time. This stability is also reflected in the *R*_corr_ values obtained, which follows the same trend. The *R*_corr_ slightly decreases upon exposure to the SBF and then remain almost constant along the immersion time (Figure 17B). In this case, the slight decrease of both *R*_coat_ and *R*_corr_ values followed by a constant value within the immersion time (Figure 17B), prove good stability and lower porosity of this coating when compared to the one obtained at 600 °C for 2 h. The high increase of the *R*_corr_ values observed in the samples coated at 600 °C/2 h is not here evidenced. Nevertheless, the evolution of the Nyquist plots with the immersion time shows a slow decrease of the diameter of the arcs and thus the *R*_corr_ values of these samples, evidencing a best behaviour against corrosion during all the immersion tests in an aggressive solution such as the SBF. The morphology of the coatings plays an important role in the different behaviour of these coatings. Moreover, in both cases, the penetration of the SBF is allowed, mainly in the upper part of the coating and thus the formation of HAp from the solution, which was one of the aims of this study. However, the kinetic of this process is different in both cases, as reflected in the impedance results obtained. The porosity of the coating obtained at 600 °C/2 h favours a rapid ingress of the electrolyte and allows that the formation of the HAp into this pores occurs in a bigger extent than in the coating obtained at 800 °C/2 h. However, on the other hand, due to this porosity the coating obtained at 600 °C/2 h shows a lesser barrier effect than the one provided for the coating obtained at 800 °C/2 h.

Table 6 shows the values obtained for the exponent n of the *CPE*_dl_ of the equivalent circuits A and B applying the CNLS fits of the ZView software to the impedance experimental plots of HAp-Coatings/Ti6Al4V systems. The *n* values are directly related to the corrosion mechanisms. *CPE*_dl_ would represent the double layer capacitance of the metal/electrolyte interface at the base of pores and perforations of the coating. For the HAp-Coatings/Ti6Al4V samples thermally treated at 800 °C/2 h the *n* values are situated into the 0.41–052 range (Table 6). These values are closer to those typically ascribed to diffusion mechanisms (*n* = 0.5), than to *n* values attributed to the electrochemical double layer capacitance (*n* = 1). From all the exposed above, it seems that the diffusion mechanisms though the pores and microcracks of the coating dominate on the activation processes and corrosion at the metal surface, preventing in this way the ion release from the metallic substrate to the SBF.

## 4. Conclusions

Hydroxyapatite crystalline coatings on Ti6Al4V alloy have been obtained by sol-gel route. The crystallite size and degree of crystallinity of the HAp sol-gel-derived coatings strongly depends on the thermal treatment applied. Based on this knowledge, the prepared HAp sol-gel coating/Ti6Al4V systems were thermally treated during 2 h at 600 °C and 800 °C, respectively. The obtained results showed that the prepared coatings are crystalline HAp with little deviations from that present in the human bone. All the prepared HAp sol-gel-derived coating/Ti6Al4V systems showed good bioactivity upon immersion in SBF for fifteen days. 3-[4,5-dimethylthiazol-2-yl]-2,5-diphenyl MTT and Alamar blue cell viability assays were used to study the biocompatibility of the resulting HAp sol-gel-derived coatings. The cell viability absorbance tests have shown that the leachate obtained during a period of 7 days showed a low toxicity for cultures of osteoblasts used as model, tending moreover to disappear any sign cytotoxicity over the period of 7 days defined in the experimental design. Both obtained HAp sol-gel-derived coatings showed strong adhesion at the coating/alloy interface. In terms of corrosion protection, the HAp coating/Ti6Al4V systems thermally treated in the range 600–800 °C exhibited good corrosion protection preventing the ion release from the Ti6Al4V alloy to the SBF.

## Figures and Tables

**Table 1:** Crystallite size and degree of crystallinity of the sol-gel-derived powders thermally treated at different temperatures.

Sample	HAp Sol-Gel-Derived Powders		
	Thermal Treatment T (°C)	Crystallite Size (nm)	Degree of Crystallinity (%)
S6	600	40	85
S8	800	51	89
S12	1200	54	82

**Table 2:** Thickness measurements of HAp sol-gel-derived coatings treated at different temperatures.

Sample	HAp Sol-Gel-Derived Coatings	
	Thermal Treatment T (°C)	Thickness (µm)
S6	600	7.74 ± 0.655
S8	800	8.51 ± 0.135
S12	1200	8.63 ± 0.121

**Table 3:** Values of shear strength in MPa of five samples tested.

Frequency	Mean	Median	Geometric Mean	Variance	Standard Deviation	Standard Error	Minimum	Maximum
5	29.036	28.94	28.8462	13.1609	3.6278	1.6224	23.54	33.35

**Table 4:** Surface roughness parameters for uncoated Ti6Al4V alloy and HAp coated Ti6Al4V alloy thermally treated at 600 °C/2 h and 800 °C/2 h.

Sample	Ra (µm)	Rp (µm)	Rv (µm)
Ti6Al4V (nude)	0.094 ± 0.018	0.467 ± 0.153	−0.361 ± 0.104
S6 (TT 600 °C/2 h)	0.163 ± 0.028	1.390 ± 0.911	−0.650 ± 0.155
S8 (TT 800 °C/2 h)	0.165 ± 0.012	0.631 ± 0.150	−0.606 ± 0.103

**Table 5:** Values obtained for the elements of the proposed equivalent circuits applying CNLS fits of the ZView software on experimental impedance plots of samples S6 (600 °C/2 h) and S8 (800 °C/2 h), respectively. Immersion time in SBF: 5 days.

Electrical Element	Equivalent Circuit A			Equivalent Circuit B		
	Value	Error	Error %	Value	Error	Error %
*R*_s_	138.8	3.43	2.47	126.6	2.95	2.33
*R*_coat_	1014.0	49.36	4.93	747.3	37.51	5.02
*CPE*_coat_-*Y*_0_	2.32 × 10^−6^	3.46 × 10^−7^	14.93	3.36 × 10^−6^	5.30 × 10^−7^	15.8
*CPE*_coat_-*n*	0.67	0.01	2.17	0.68	0.02	2.56
*CPE*_dl_-*Y*_0_	7.05 × 10^−5^	5.99 × 10^−7^	0.85	7.16 × 10^−5^	5.69 × 10^−7^	0.79
*CPE*_dl_-*n*	0.46	4.00 × 10^−3^	0.86	0.46	4.59 × 10^−3^	0.99
*R*_corr_	1.46 × 10^5^	6.99 × 10^3^	4.77	1.51 × 10^5^	7.96 × 10^3^	5.27
Chi-Squared (χ^2^)	0.0009			0.0012		

**Table 6:** *CPE*_dl_-*n* values and % of relative error obtained with the equivalent circuits A and B applying CNLS fits from the ZView software on experimental impedance plots of samples S6 (600 °C/2 h) and S8 (800 °C/2 h), for variable immersion time in SBF.

Immersion Time in SBF	*CPE*_dl_-*n* Values							
	Sample S6 (TT: 600 °C/2 h)				Sample S8 TT: 800 °C/2 h			
	Equivalent Circuit A		Equivalent Circuit B		Equivalent Circuit A		Equivalent Circuit B	
	Value	Error %	Value	Error %	Value	Error %	Value	Error %
5 min	0.525	0.96	0.613	5.61	0.455	1.97	0.476	3.11
1 day	9.268	9.35	0.628	1.18	0.415	0.80	0.410	1.22
2 days	1.933	8.31·10^3^	0.624	21.9	0.422	0.77	0.423	1.02
5 days	0.651	5.99	0.644	3.43	0.464	0.86	0.463	0.99
9 days	0.491	2.96	0.513	1.47	0.477	0.92	0.478	1.05
15 days	0.630	1.20	0.636	0.98	0.511	1.03	0.516	1.10
